# HMGB1 Derived from the Pyroptotic Microenvironment Promotes Macrophage Extracellular Traps in Hirschsprung‐Associated Enterocolitis

**DOI:** 10.1002/adbi.202400761

**Published:** 2025-06-04

**Authors:** Rui Zhang, Jing Li, Lili Song, Liya Pan, Chengchen Zhang, Zhiyan Zhan, Li Hong

**Affiliations:** ^1^ Department of Clinical Nutrition Shanghai Children's Medical Center Shanghai Jiao Tong University School of Medicine 1678 Dongfang Road Shanghai 200127 China; ^2^ Department of Hematology & Oncology Key Laboratory of Pediatric Hematology and Oncology Ministry of Health Shanghai Children's Medical Center Shanghai Jiao Tong University School of Medicine 1678 Dongfang Road Shanghai 200127 China; ^3^ Clinical Research Center Shanghai Children's Medical Center Shanghai Jiao Tong University School of Medicine 1678 Dongfang Road Shanghai 200127 China

**Keywords:** high mobility group box 1, hirschsprung‐associated enterocolitis, macrophage, macrophage extracellular traps, pyroptosis

## Abstract

Hirschsprung‐associated enterocolitis (HAEC) is the most common and severe complication in patients with Hirschsprung's disease (HSCR) and is characterized by high morbidity, frequent recurrence and substantial mortality. The formation of macrophage extracellular traps (METs), a novel inflammatory mode of cell death, plays a significant role in the progression of various inflammatory diseases. However, the mechanisms underlying METs formation and their role in the progression of HAEC remain unclear. Here, the findings indicate that METs formation induced by the pyroptotic microenvironment enhances inflammatory responses and induces colonic epithelial cells (CECs) injury in HAEC. Mechanistically, high mobility group box 1 protein (HMGB1), derived from this pyroptotic environment, mediates METs formation through toll‐like receptor 4 (TLR4)‐p38 MAPK/p65 NF‐kB signaling pathways. Furthermore, incubation of CECs with METs induces suppression of cell viability, more production of reactive oxygen species (ROS) and pyroptosis. In conclusion, HMGB1 mediates the communication between pyroptotic microenvironment and METs formation, triggering enhanced inflammatory responses and damage to CECs. Targeting HMGB1 presents a potential therapeutic strategy for HAEC.

## Graphical abstract

HMGB1 derived from the pyroptotic environment in Hirschsprung‐associated enterocolitis mediates the formation of macrophage extracellular traps through TLR4 ‐p38 MAPK/p65 NF‐kB signaling pathways. Macrophage extracellular traps induce increased ROS production and pyroptosis of colonic epithelial cells. Targeting the formation of macrophage extracellular traps by inhibiting TLR4‐p38 MAPK/p65 NF‐kB pathways is a potential therapeutic strategy for Hirschsprung‐associated enterocolitis.

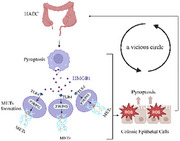

## 1 Introduction

Hirschsprung's disease (HSCR) is a developmental abnormality that affects intestinal movement.^[^ ^1^ ^]^ Among its complications, Hirschsprung‐associated enterocolitis (HAEC) stands out as the most prevalent and severe condition.^[^ ^2^ ^]^ Previous studies suggest that HAEC occurrence ranges from 20% to 58%, with recurrence observed in half of the cases, while mortality rates vary between 1% and 30%.^[^ ^3^ ^]^ Consequently, there is an urgent need to elucidate the underlying mechanisms and pathological signaling pathways involved in HAEC in order to develop effective strategies for prevention and therapeutic intervention.

Macrophages serve as primary defenders within the mucosal region and play critical roles in preserving intestinal equilibrium and regulating inflammatory processes in gut mucosa.^[^ ^4^ ^]^ Previous studies by our and other research groups have documented significant infiltration of pro‐inflammatory M1 macrophages in the diseased colon of patients suffering from HAEC.^[^ ^5^ ^]^ And there exists a vicious circle, where *Veillonella.parvula* (*V. parvula*)‐induced macrophage inflammation via LPS‐TLR4 pathway impairs intestinal movement, thereby exacerbating gut microbiota dysbiosis and facilitating further progression of HAEC. Disrupting this harmful cycle by inhibiting macrophage activation may represent a promising therapeutic strategy for individuals afflicted with HAEC.^[^ ^6^ ^]^ Thus, macrophages emerge as essential mediators within the intestinal immune mechanisms associated with HAEC.

Recently, accumulating evidences have started to support the notion that macrophages play a significant role in the progression of inflammatory diseases through the formation of macrophage extracellular traps (METs), which are composed of extracellular chromatin decorated with citrulline histone H3 (citH3) together with antimicrobial protein granules and enzymes.^[^ ^7^ ^]^ Since their initial characterization as a mechanism for trapping and eliminating microbes, METs have also been implicated in substantial crosstalk with various cell death pathways, contributing to the advancement of several disorders, including acute kidney injury, colon cancer, and autoimmune arthritis.^[^ ^8^ ^]^ However, direct evidence supporting the involvement of the pyroptotic microenvironment in MET formation during HAEC remains insufficient. Additional investigation is also necessary to elucidate the role of METs in orchestrating intestinal mucosal immunity.

In this study, we identified an unrecognized pathogenic role for MET formation in regulating intestinal mucosal immunity during HAEC. We demonstrated that elevated levels of METs are associated with deteriorated pathological conditions observed in colon tissues from HAEC patients and model mice. Moreover, we confirmed that high mobility group box 1 protein (HMGB1) derived from the pyroptotic microenvironment activated the p38 MAPK/P65 NF‐kB signaling cascade via Toll‐like receptor 4 (TLR4), leading to an enhanced release of METs. Additionally, METs induce damage to colonic epithelial cells (CECs), thereby contributing to the inflammatory process. Consequently, this study provides new insights into novel mechanisms underlying MET formation during HAEC and suggests potential therapeutic targets for intervention in this condition.

## 2 Results

### 2.1 More METs Accumulate in HAEC, and the Level of MET Formation is Correlated with the Pathological Condition of Colon Tissues

First, we aimed to investigate the potential involvement of METs in the onset of HAEC in both patients and model mice. In our previous studies, we identified that the overabundance of *V. parvula* was a significant characteristic of the microbiome pattern within the intestinal flora of HAEC patients.^[^ ^9^ ^]^ Furthermore, model mice fed with LPS derived from *V. parvula* (LPS‐V) could closely simulate the clinical and pathological features associated with HAEC.^[^ ^6^ ^]^ Therefore, we initially employed histological methods to locate MET occurrence in colon tissues obtained from HAEC patients and LPS‐V‐fed mice, in order to examine whether METs are implicated in the onset of HAEC.

**Figure 1:**
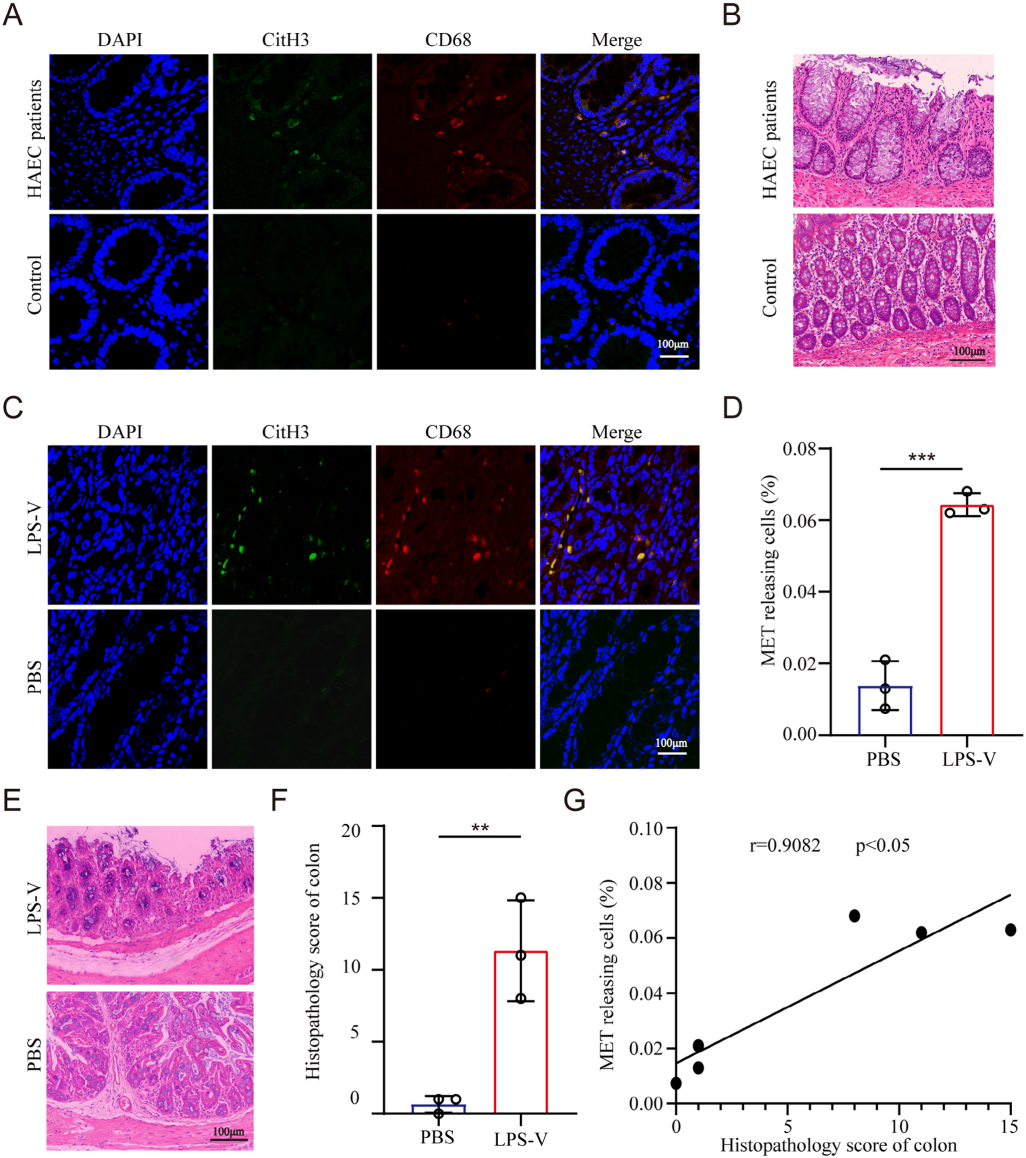
More METs accumulate in HAEC, and the level of MET formation is correlated with the pathological condition of colon tissues. A) The formation of MET in colon tissues from HAEC patients and healthy control subjects was assessed by CD68 and citH3 staining using confocal microscopy. Scale bars: 100µm. B)The injury degree of colon tissues from HAEC patients and healthy subjects was evaluated by H&E staining. Scale bars: 100µm. C,D) Colon tissues from LPS‐V‐fed model mice and control mice were collected and the formation of MET was detected with CD68 and citH3 staining using confocal microscopy. Scale bars: 100µm. The representative images are shown in C, and quantification of C is shown in D (Unpaired t‐test, *n* = 3 per group). E,F) The injury degree of colon tissues from LPS‐V‐fed model mice and control mice was detected by H&E staining. The representative images are shown in E, and histopathological scores of E is shown in F (Unpaired t‐test, *n* = 3 per group). Scale bars: 100µm. G) Correlation between METs levels and histopathological scores in colon tissues of mice from LPS‐V‐fed group and control group. (Pearson correlation coefficient, *n* = 3 per group). Data are expressed as mean ± SD. **p < 0.01; ***p < 0.001.

Immunofluorescence staining of CD68 and citrulline histone H3 (citH3) was performed to assess MET formation. We detected elevated MET levels in the colon tissues from HAEC patients and LPS‐V‐fed mice, indicating significant MET deposition (**Figure** **1**A,C,D). Additionally, colon tissues from HAEC patients and LPS‐V‐fed mice exhibited severe pathology characterized by extensive epithelial damage (Figure 1B,E,F). Notably, we further observed that increased levels of METs correlated strongly with more severe pathological conditions in the colon tissues of model mice (Figure 1G).

Together, these findings suggest that METs accumulate in the colon tissues of both HAEC patients and LPS‐V‐fed mice and correlate with adverse pathological conditions.

### 2.2 The Pyroptotic Microenvironment Recruits Macrophages and Induces MET Formation

As shown above, we observed a correlation between the levels of METs and poor histopathological scores in LPS‐V‐fed mice. It has been extensively established that the crosstalk between pyroptosis and the formation of immune cell extracellular traps contributes to the progression of various diseases, including acute respiratory distress syndrome (ARDS), acute liver injury, and gastric cancer.^[^ ^10^ ^]^ In this study, we sought to investigate the relationship between pyroptosis and MET formation in HAEC.

**Figure 2:**
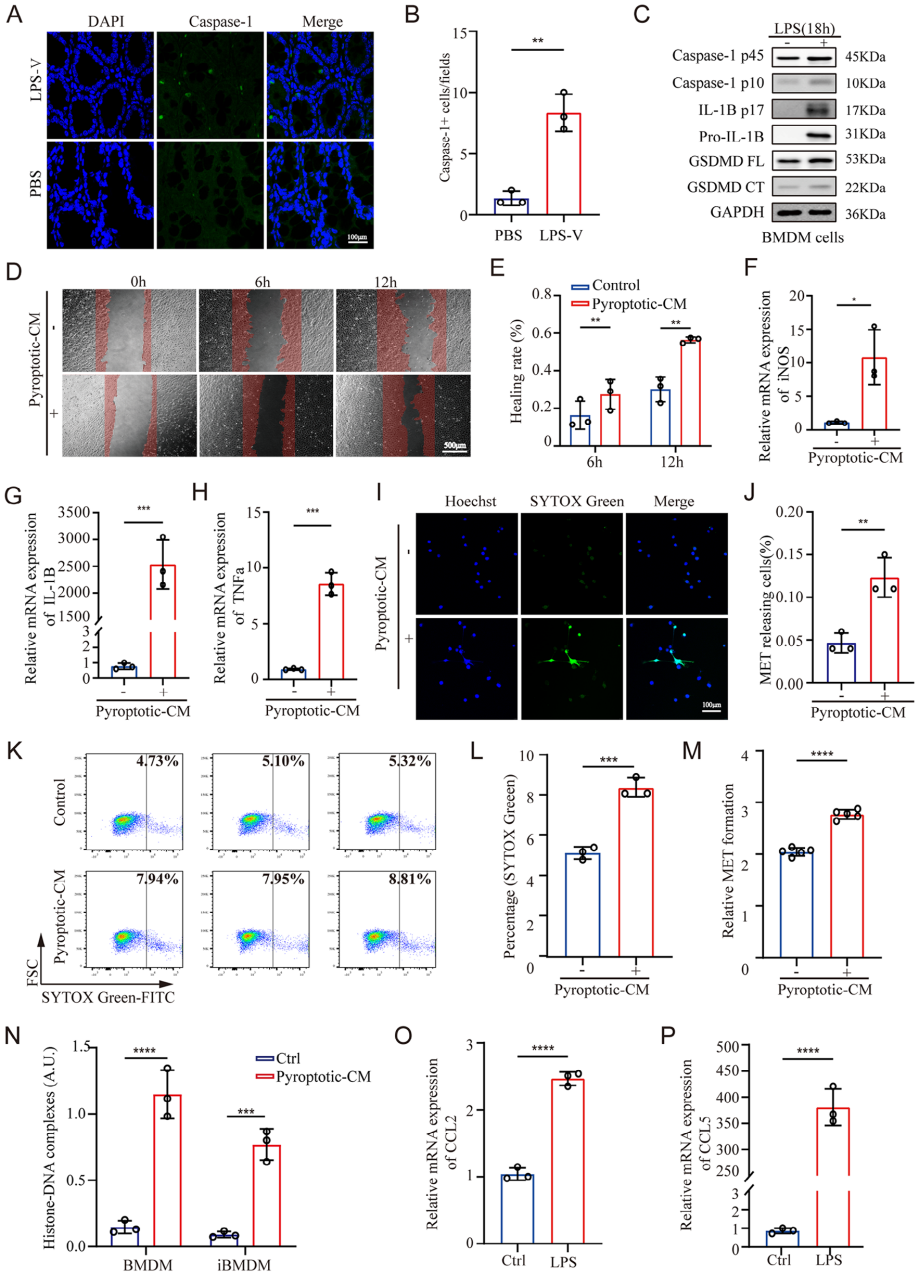
The pyroptotic microenvironment recruits macrophages and induces MET formation. A,B) The level of pyroptosis in colon tissues from LPS‐V‐fed model mice and control mice was assessed by caspase‐1 staining and detected by confocal microscopy. The representative images are shown in A and the quantification of A is shown in B (Unpaired t‐test, *n* = 3 per group). Scale bars: 100µm. C) Western blot analysis was performed to evaluate the expression of full‐length and cleaved fragment of caspase‐1, IL‐1β, and GSDMD proteins in lysates of BMDMs treated with LPS. D,E) The migration of macrophages incubated with or without pyroptotic‐CM was assessed by wound healing assays. The representative images are shown in D and the quantification of D is shown in E (Unpaired t‐test, *n* = 3 per group). Scale bars: 500µm. F‐H) BMDMs were incubated with or without pyroptotic‐CM, and the levels of markers indicative of M1‐polarized macrophages, including iNOS, IL‐1β, and TNF‐α, were measured using qRT‐PCR (Unpaired t‐test, *n* = 3 per group). I,J) Macrophages were stimulated by pyroptotic‐CM to induce MET formation that was confirmed by SYTOX green staining and detected by confocal microscopy. The representative images are shown in I and the quantification of I is shown in J (Unpaired t‐test, *n* = 3 per group). Scale bars: 100µm. K,L) Macrophages were incubated with pyroptotic‐CM and the MET formation was assessed by SYTOX green staining and detected by flow cytometry. The representative images are shown in K and the quantification of K is shown in L (Unpaired t‐test, *n* = 3 per group). M) Quantification of MET formation in the supernatant of macrophages stimulated with pyroptotic‐CM was assessed by SYTOX Green staining and detected by microplate reader (Unpaired t‐test, *n* = 5 per group). N) The level of histone‐DNA complexes in METs isolated from macrophages stimulated with pyroptotic‐CM or not were detected with ELISA (Unpaired t‐test, *n* = 3 per group). O,P) BMDMs were treated with or without LPS, and the levels of macrophage chemotactic factors, CCL2 and CCL5, were measured using qRT‐PCR (Unpaired t‐test, *n* = 3 per group). Data are expressed as mean ± SD. *p < 0.05, **p < 0.01, ***p < 0.001, ****p < 0.0001.

Initially, we detected a significant increase in caspase‐1 expression within colon tissues from LPS‐V‐fed model mice through immunofluorescence analysis, indicating the onset of pyroptosis (**Figure** **2**A,B). Since previous studies had confirmed the increased accumulation of macrophages in individuals with HAEC compared to healthy controls,^[^ ^5^ ^]^ we aimed to explore the role of the pyroptotic microenvironment in promoting macrophage accumulation and MET formation.

A cellular model of pyroptosis was established for further investigation. The pyroptosis of macrophages was induced through LPS transfection, a common approach that is widely acknowledged for triggering pyroptosis.^[^ ^11^ ^]^ The bone marrow‐derived macrophages (BMDMs) were collected at day 6 post‐differentiation and stimulated in vitro through LPS transfection to induce pyroptosis. The occurrence of pyroptosis was validated via western blotting as LPS treatment resulted in enhanced cleavage of IL‐1β, caspase‐1, and GSDMD into their mature forms (Figure 2C). Next, the pyroptotic‐conditioned medium (pyroptotic‐CM) from pyroptotic BMDMs was collected to simulate the pyroptotic microenvironment for subsequent experiments. Our findings demonstrated that treatment with pyroptotic‐CM significantly enhanced the migratory capacity of these macrophages (Figure 2D,E). Furthermore, exposure to pyroptotic‐CM markedly increased the mRNA levels of iNOS, TNF‐α, and IL‐1β, which are markers indicative of M1‐polarized macrophages (Figure 2F–H). Additionally, incubation with pyroptotic‐CM significantly enhanced the formation of METs, as evaluated through SYTOX Green staining and measured using fluorescence microscopy, flow cytometry and fluorometry (Figure 2I–M).In accordance with the aforementioned findings, the level of histone‐DNA complexes released by macrophages incubated with pyroptotic‐CM was significantly higher than control group (Figure 2N). Interestingly, the mRNA expression levels of CCL2 and CCL5, which are classical macrophage chemokines, were found to be elevated in pyroptotic cells, suggesting a potential role for the pyroptotic microenvironment in the recruitment of macrophages (Figure 2O,P). Notably, this finding aligns with the results from the wound healing assay described above. Collectively, these findings suggest that the pyroptotic microenvironment plays a crucial role in promoting the MET formation. The crosstalk between pyroptosis and MET formation may establish a vicious cycle that exacerbates inflammation, thereby facilitating the progression of HAEC.

### 2.3 HMGB1 Derived from the Pyroptotic Microenvironment Induces MET Formation

HMGB1, a non‐histone nuclear protein, typically operates as a DAMP molecule upon active or passive liberation into the extracellular space, which orchestrates inflammatory and immune processes via various receptors or direct cellular uptake.^[^ ^12^ ^]^ Prior research indicates that HMGB1 serves as a signaling intermediary that facilitates intercellular communication during immune responses and has been implicated in the progression of various diseases, including thrombosis, sepsis, and lung cancer.^[^ ^13^ ^]^ However, whether HMGB1 participates in the pathophysiological processes of HAEC and its underlying mechanisms remain unclear.

**Figure 3:**
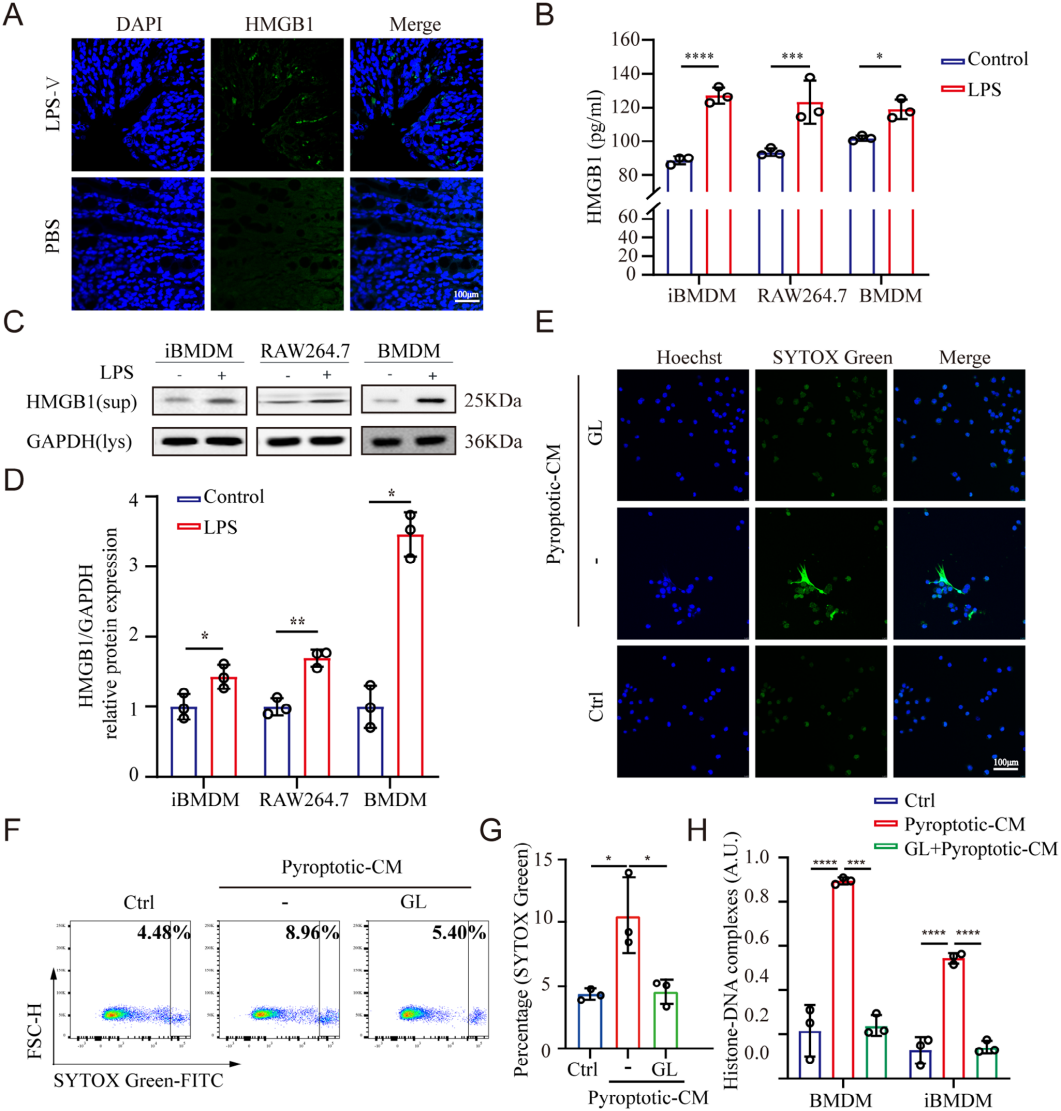
HMGB1 derived from the pyroptotic microenvironment induces MET formation. A) The expression of HMGB1 in colon tissues from LPS‐V‐fed model mice and control mice was evaluated by IF staining. Scale bars: 100µm. B) The level of HMGB1 in the supernatant of macrophages stimulated with LPS (1µg mL^−1^) for 18h was assessed by ELISA (Unpaired t‐test, *n* = 3 per group). C,D) Immunoblot analysis of HMGB1 protein in supernatant of BMDMs treated with LPS (1µg mL^−1^) for 18h. The representative images are shown in C and the quantification of C is shown in D (Unpaired t‐test, *n* = 3 per group). E) Macrophages were pretreated with or without GL (HMGB1 inhibitor, 50µM), and then were stimulated by pyroptotic‐CM to induce MET formation that was confirmed by SYTOX green staining and detected by confocal microscopy. Scale bars: 100µm. F,G) Macrophages were stimulated by pyroptotic‐CM with or without GL (50µM), and the MET formation was assessed by flow cytometry. The representative images are shown in F and the quantification of F is shown in G (one‐way ANOVA followed by Tukey's multiple‐comparison test, *n* = 3 per group). H) Histone‐DNA complexes levels in METs isolated from macrophages stimulated by pyroptotic‐CM with or without GL (50µM) were detected with ELISA (one‐way ANOVA followed by Tukey's multiple‐comparison test, *n* = 3 per group). Data are expressed as mean ± SD. *p < 0.05, **p < 0.01, ***p < 0.001, ****p < 0.0001.

In order to examine whether HMGB1 is involved in the onset of HAEC, the expression of HMGB1 was detected by immunofluorescence within colon tissues from LPS‐V‐fed model mice and control mice. The results indicated a significant upregulation of HMGB1 expression in the colon tissues of LPS‐V‐fed model mice compared to control mice, suggesting a clinical relevance between HMGB1 and the pathogenesis of HAEC (**Figure** **3**A). Next, we want to elucidate the role of HMGB1 in the crosstalk between the pyroptotic microenvironment and MET formation during HAEC. We detected whether the pyroptotic‐CM contains HMGB1, which was measured by western blotting and ELISA. The results showed that the level of HMGB1 in the pyroptotic‐CM of macrophages was significantly elevated compared to the control group, which was corroborated across three distinct cell lines (Figure 3B–D). It is widely proven that Glycyrrhizin (GL), a natural compound extracted from Glycyrrhiza glabra (licorice plant), exhibits the capacity to suppress HMGB1 secretion and prevent extracellular HMGB1 from interacting with its receptors.^[^ ^14^ ^]^ Macrophages were treated with pyroptotic‐CM for MET induction both with and without the HMGB1 antagonist GL to specifically explore the role of HMGB1 in mediating the crosstalk between the pyroptotic microenvironment and MET formation. The formation of MET was evaluated with SYTOX Green staining using fluorescence microscopy and flow cytometry, and the results indicated that the inhibition of HMGB1 significantly attenuates MET formation induced by pyroptotic‐CM compared to groups treated without the HMGB1 inhibitor (Figure 3E–G). In alignment with the aforementioned findings, treatment of macrophages with GL prior to incubation with pyroptotic‐CM resulted in a reduction in the release of histone‐DNA complexes (Figure 3H).

Taken together, our findings demonstrate a significant upregulation of HMGB1 expression within colon tissues from LPS‐V‐fed model mice, which serves as a critical mediator in the interaction between the pyroptotic microenvironment and MET formation. Therefore, targeting HMGB1 may represent an effective therapeutic strategy for HAEC.

**Figure 4:**
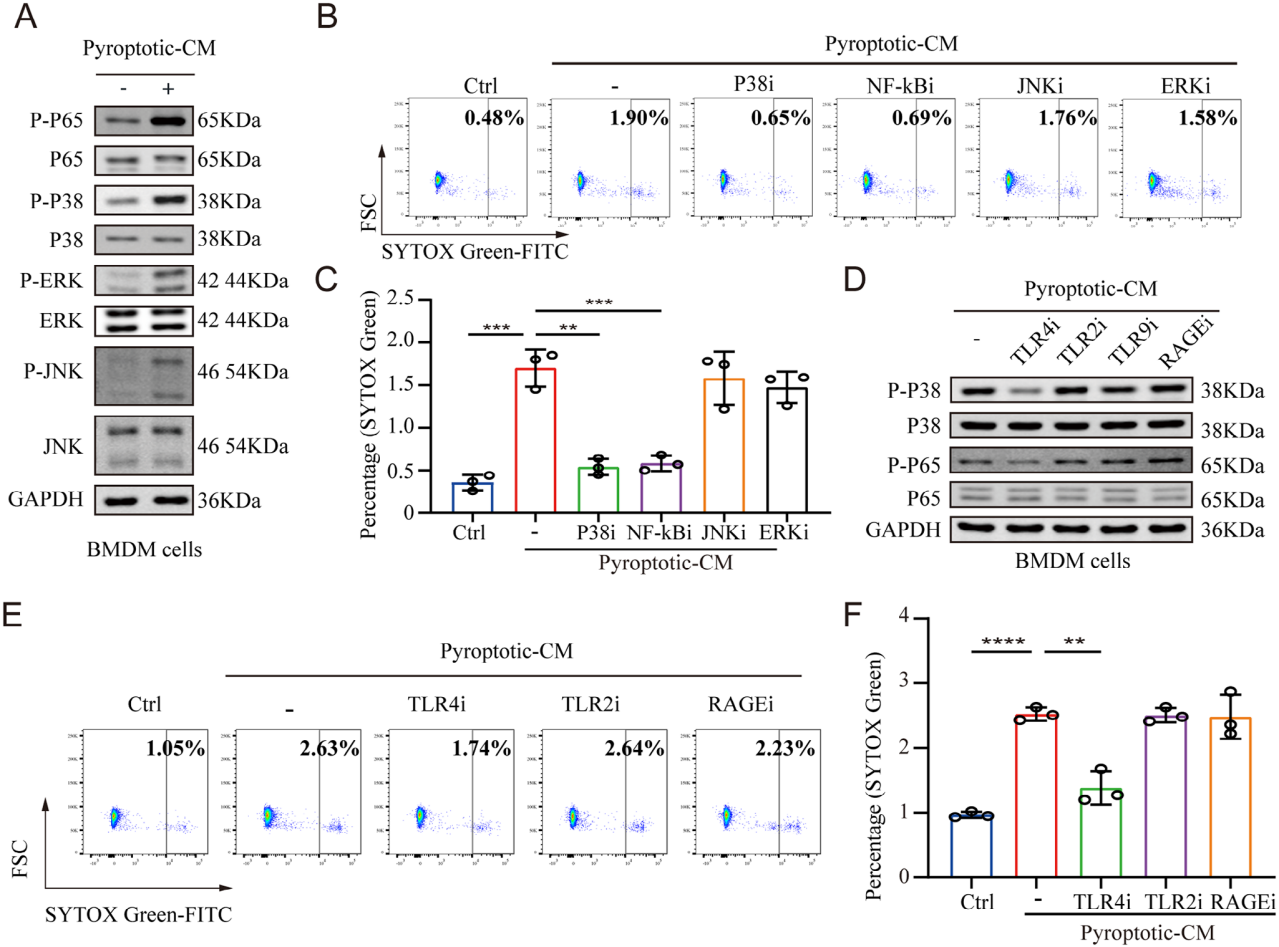
The TLR4‐P38 MAPK/P65 NF‐kB signaling pathways mediate the‐pyroptotic‐microenvironment‐induced MET formation. A) Western blot analysis of p‐ERK, p‐p38, p‐JNK and p‐p65 levels in macrophages cocultured with pyroptotic‐CM for 4 h. B,C) Macrophages were pretreated with inhibitors of the ERK, JNK, p38 MAPK, and p65 NF‐kB pathways prior to incubation with pyroptotic‐CM. The MET formation by macrophages was evaluated with SYTOX Green staining and detected by flow cytometry. The representative images are shown in B and the quantification of B is shown in C (one‐way ANOVA followed by Tukey's multiple‐comparison test, *n* = 3 per group). D) Macrophages were pretreated with inhibitors of TLR2, TLR4, TLR9 and RAGE prior to coculture with pyroptotic‐CM, and the p‐p38 and p‐p65 levels in macrophages were measured by western blotting. E,F) The MET formation of macrophages pretreated with inhibitors of TLR2, TLR4 and RAGE prior to incubation with pyroptotic‐CM was evaluated with SYTOX Green staining and detected by flow cytometry. The representative images are shown in E and the quantification of E is shown in F (one‐way ANOVA followed by Tukey's multiple‐comparison test, *n* = 3 per group). Data are expressed as mean ± SD. **p < 0.01, ***p < 0.001, ****p < 0.0001.

### 2.4 The TLR4‐P38 MAPK/P65 NF‐kB Signaling Pathways Mediate the‐Pyroptotic‐Microenvironment‐Induced MET Formation

To clarify the mechanism by which METs are released in the pyroptotic microenvironment, BMDMs were treated with pyroptotic‐CM or PBS. The findings indicated that pyroptotic‐CM activated the NF‐kB and MAPK signaling cascades (**Figure** **4**A). Subsequently, BMDMs were pretreated with specific inhibitors targeting the p65, p38, ERK, or JNK pathways prior to exposure to pyroptotic‐CM. Among the various signaling pathway blockers tested, pretreatment with p38 and p65 inhibitors exhibited the most substantial reduction in MET generation, indicating that both the p38 MAPK and p65 NF‐kB pathways are essential for MET formation induced by the pyroptotic microenvironment (Figure 4B,C).

**Figure 5:**
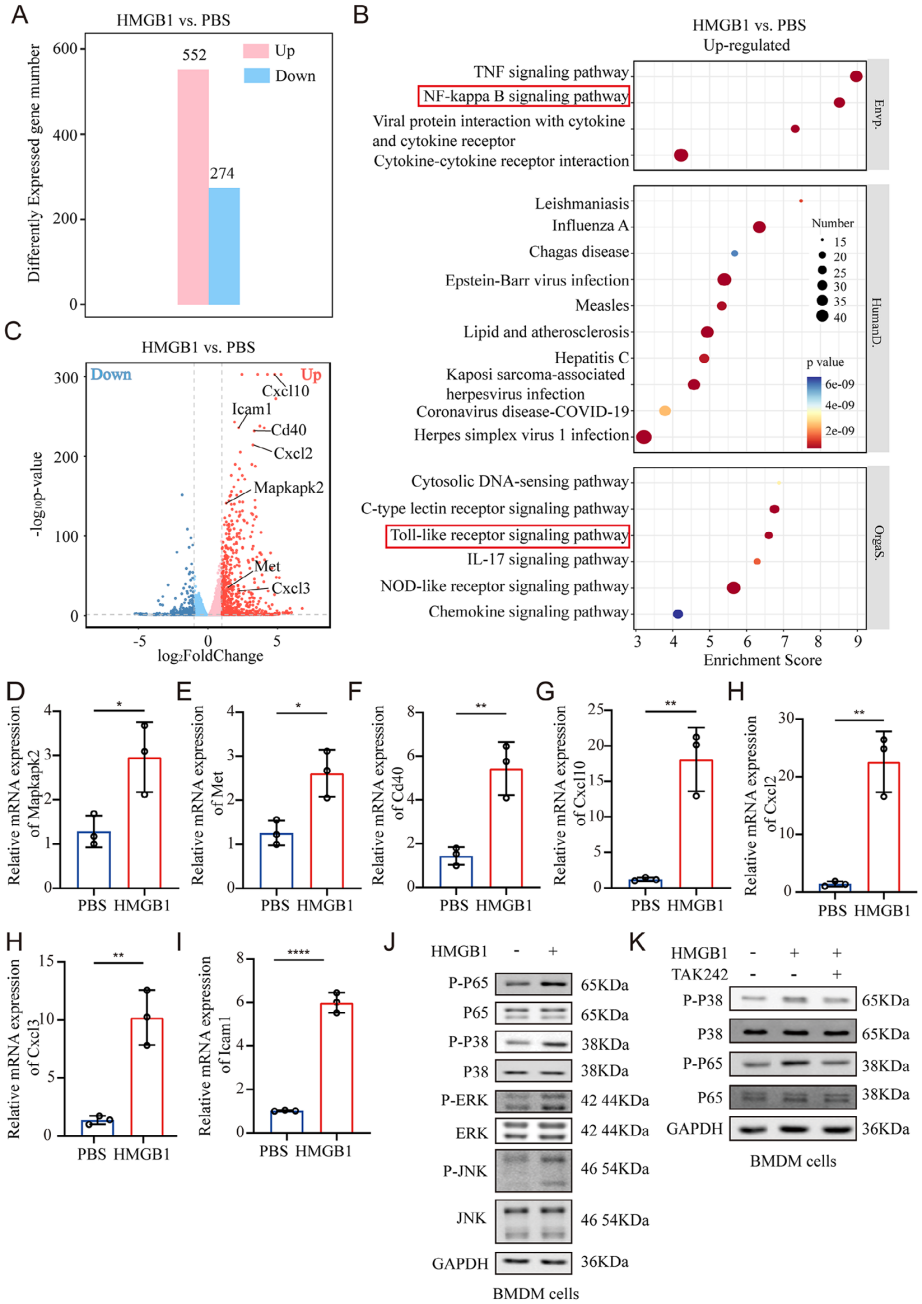
HMGB1 induces MET formation through TLR4‐P38 MAPK/P65 NF‐kB signaling pathways in macrophages. A‐C) BMDMs isolated from mice were stimulated with HMGB1 or PBS. Then, differentially expressed genes (DEGs) were analyzed by RNA sequencing. (A) The number of DEGs in the HMGB1 group vs. the PBS group. Red represented upregulated DEGs, and blue downregulated DEGs. (B) KEGG (Kyoto Encyclopedia of Genes and Genomes) pathway enrichment analyses of the upregulated DEGs. The dot size represents the number of DEGs, and the dot color represents the corresponding p value. (C) Scatter plot showing DEGs in the HMGB1 group vs. the PBS group. Genes were plotted based on their expression levels. Red and green dots represented up and downregulated genes, respectively. D‐I) qRT‐PCR analysis of the indicated genes in macrophages treated with HMGB1 or PBS (Unpaired t‐test, *n* = 3 per group). J) Western blot analysis of p‐ERK, p‐p38, p‐JNK and p‐p65 levels in macrophages cocultured with HMGB1. K) Macrophages were pretreated with inhibitors of TLR4 prior to incubation with HMGB1, and the p‐p38 and p‐p65 levels in macrophages was measured by western blotting. Data are expressed as mean ± SD. *p < 0.05, **p < 0.01, ****p < 0.0001.

Given that HMGB1 in the extracellular space substantially promotes MET formation under pyroptotic conditions, we investigated the subsequent signaling cascades of HMGB1 during MET development. Previous studies have established that toll‐like receptors (TLRs) and the receptor for advanced glycation end products (RAGE) serve as crucial HMGB1 receptors in inflammatory environments,^[^ ^15^ ^]^ however the specific receptor mediating the interactions between pyroptosis and MET formation remains unclear. Therefore, we administered TLR2, TLR4 or RAGE inhibitors to BMDMs before exposing them to the pyroptotic‐CM. It was observed that only the TLR4 inhibitor‐rather than TLR2 or RAGE inhibitors‐abolished activation of both p38 MAPK and p65 NF‐kB pathways as well as subsequent MET formation induced by pyroptotic‐CM (Figure 4D–F).

In summary, our findings reveal that the pyroptotic microenvironment triggers MET formation via TLR4‐p38 MAPK/p65 NF‐kB signaling pathways in HAEC.

### 2.5 HMGB1 Induces MET Formation Through TLR4‐P38 MAPK/P65 NF‐kB Signaling Pathways in Macrophages

To further elucidate the molecular mechanism of HMGB1 in inducing MET formation, BMDMs isolated from mice were stimulated with either HMGB1 or PBS and their mRNA expression profiles were analyzed by RNA sequencing. By comparing the transcriptional characteristics of BMDMs treated as described above, we identified 552 genes upregulated by at least two‐fold in BMDMs treated with HMGB1 (**Figure** **5**A). Furthermore, pathway enrichment analysis based on the Kyoto Encyclopedia of Genes and Genomes (KEGG) revealed significant activation of NF‐kB and Toll‐like receptor pathways (Figure 5B).

Subsequently, a detailed examination of the RNA‐Seq data indicated that numerous genes associated with TLR, NF‐kB, and MAPK pathways exhibited significant alterations in expression following treatment with HMGB1 (Figure 5C). Notably, several key genes‐including Met and Mapkapk2 involved in the MAPK pathway; Cd40 and Cxcl10 related to the TLR pathway; as well as Cxcl2, Cxcl3, and Icam1 linked to the NF‐kB pathway‐were found to be upregulated. This finding was corroborated by qRT‐PCR analyses (Figure 5D–I). Collectively, these results suggest that HMGB1 may induce MET formation via TLR‐MAPK/NF‐kB signaling pathways.

To further validate this mechanism, BMDMs were treated with HMGB1 or PBS. The findings demonstrated that HMGB1 activated both NF‐kB and MAPK pathways within BMDMs, while pretreatment with a TLR4 inhibitor effectively abrogated these activated pathways (Figure 5J,K). This observation aligns with our understanding of how METs are released under pyroptotic microenvironments.

In summary, these evidences indicate that HMGB1, a critical component of the pyroptotic microenvironment, induces MET formation through TLR4‐P38 MAPK/P65 NF‐kB signaling pathways during HAEC.

### 2.6 MET Formation Enhances Inflammatory Responses and Induces Damage to CECs

**Figure 6:**
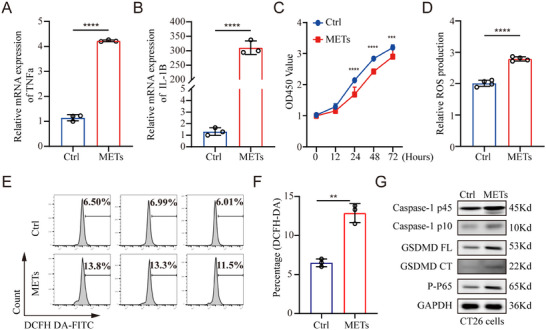
MET formation enhances inflammatory responses and induces damage to CECs. A,B) BMDMs were treated with pyroptotic‐CM to induce MET formation, followed by co‐incubating with WT untreated BMDM. TNF‐α and IL‐1β mRNA levels in the WT BMDM were then measured by qRT‐PCR (Unpaired t‐test, *n* = 3 per group). C) CT26 cells were treated with METs or PBS for 0, 12, 24, 48 and 72 h, after which cell viability was detected by CCK‐8 assays (two‐way ANOVA followed by Sidak's multiple‐comparison test, *n* = 6 per group). D) CT26 cells were stimulated with METs or PBS and the ROS production was measured with DCFH‐DA staining and detected by microplate reader (Unpaired t‐test, *n* = 4 per group). E,F) Levels of ROS in CT26 cells after treatment of METs or PBS were assessed by DCFH‐DA staining and detected by flow cytometry. The images of flow cytometry are shown in E and the quantification of E is shown in F (Unpaired t‐test, *n* = 3 per group). G) Immunoblot analysis of p‐p65, GSDMD, and caspase‐1 protein in lysates of CT26 cells treated with METs. Data are expressed as mean ± SD. **p < 0.01, ***p < 0.001, ****p < 0.0001.

To elucidate the role of METs in inflammatory responses, we initially treated macrophages with pyroptotic‐CM to induce MET formation. Subsequently, BMDMs were incubated with the METs to evaluate their impact on the inflammatory process. The relative mRNA levels of TNF‐α and IL‐1β showed a significant increase in macrophages exposed to METs compared to those treated with PBS (**Figure** **6**A,B).

Given that elevated levels of METs have been observed in clinical specimens from individuals with HAEC, we aimed to investigate whether METs cause damage to CECs. A new cell line, CT26, was introduced; this line is commonly used as a model for colonic epithelial cells to study intestinal barrier damage induced by various physicochemical factors. The cell viability of CT26 cells was markedly reduced following incubation with METs for 24, 48, and 72 h (Figure 6C). Furthermore, we explored the mechanisms through which METs induce damage in CECs. Our findings revealed that exposure to METs led to increased reactive oxygen species (ROS) production as well as pyroptosis in CECs (Figure 6D–G).

Overall, our results indicate that MET formation enhances inflammatory responses and acts as a mediator in the interaction between colonic epithelial cells and macrophages, thereby contributing further to the progression of HAEC.

## 3 Discussion

HAEC, the most significant and severe complication associated with HSCR, is characterized by a notably elevated incidence, frequent recurrence, and substantial mortality rates, establishing it as a critical public health concern.^[^ ^1^, ^2^ ^]^ Further investigation into the mechanisms and effective therapeutic targets of HAEC have become a common objective among researchers. Macrophages, the primary inflammatory cells in the gut, have been extensively confirmed to be activated and involved in HAEC.^[^ ^16^ ^]^ However, the mere presence of infiltrated activated macrophages does not fully account for the extensive systemic inflammatory response observed in HAEC. Moreover, emerging research suggests that crosstalk between pyroptosis and immune cell extracellular traps plays a crucial role in various disorders such as ARDS and cancer,^[^ ^[Link]^, ^[Link]^ ^]^ leading us to hypothesize whether there exists an interaction between pyroptosis and immune cell extracellular traps that serves a crucial role during HAEC. Nevertheless, direct evidence regarding the crosstalk between pyroptotic processes and the formation of immune cell extracellular traps, along with its contribution to dysregulated inflammatory suppression present in HAEC, remains insufficient.

In this investigation, we observed that the levels of METs in patients with HAEC and LPS‐V‐fed model mice were significantly elevated compared to healthy individuals and unmodeled mice. Additionally, MET levels exhibited a positive correlation with the severity of pathological conditions in colon tissues from model mice. Experiments in vitro demonstrated that HMGB1 derived from the pyroptotic environment acted through TLR4, thereby initiating the p38 MAPK/P65 NF‐kB signaling pathway, which subsequently induced the release of METs. Furthermore, our data indicated that METs induced damage to CECs via increased ROS production and pyroptosis, suggesting the potentially crucial role of METs in epithelial barrier injury during HAEC. In conclusion, our findings reveal a previously unidentified mechanism of cell communication wherein the pyroptotic microenvironment triggers the formation of METs that amplify inflammatory responses and induce injury to CECs during HAEC. In general, our study may indicate a promising direction for therapeutic intervention of HAEC.

Inflammatory activation and subsequent death of macrophages are recognized as key initiators of HAEC.^[^ ^5^, ^16^, ^17^ ^]^ However, the precise mechanisms underlying cellular demise and their implications for HAEC development remain to be fully elucidated. Notably, the pyroptosis of macrophages has been shown to contribute to intestinal barrier damage and is considered a critical factor in colitis,^[^ ^18^ ^]^ whereas the role of MET formation in the progression of HAEC remains unclear. In this study, we observed elevated levels of MET formation in the colon tissues of both HAEC patients and model mice, which were found to correlate positively with pathological scores. These findings suggest a significant involvement of METs in the progression of HAEC and fill in a gap in our understanding regarding the role that METs play in regulating intestinal mucosal immunity.

In recent years, a growing body of studies have elucidated significant interactions among various cellular death mechanisms, particularly between pyroptosis and the formation of extracellular traps. These interactions facilitate the progression of various diseases, including ARDS, acute liver injury, and gastric cancer.^[^ ^10^ ^]^ However, direct evidence regarding the crosstalk between pyroptosis and MET formation in HAEC remains insufficient. It has been widely reported that cells undergoing pyroptosis release numerous damage‐associated molecular patterns (DAMPs), which encompass mitochondrial DNA, nuclear DNA fragments, and HMGB1, among others.^[^ ^19^ ^]^ Furthermore, HMGB1 serves as a characteristic danger signal that enhances intercellular communication and plays a crucial role in the initiation, progression, and resolution of various chronic inflammatory and autoimmune disorders.^[^ ^23^ ^]^ In this study, we investigated the potential impact of HMGB1 derived from the pyroptotic microenvironment on METs formation. Our findings suggest that there may be crosstalk between pyroptosis and MET formation during HAEC processes mediated by HMGB1. This crosstalk could establish a vicious cycle that enhances inflammatory mediator release and induces intestinal barrier damage. Elevated levels of HMGB1 have been documented in various pathological conditions, including sepsis, vascular endothelial cell injury, and cancer.^[^ ^10^, ^11^ ^]^ Research has demonstrated that HMGB1 serves a role beyond merely indicating cellular injury; it acts as a significant trigger for inflammatory cascades. Following either active secretion or passive release, extracellular HMGB1 typically functions as a DAMP molecule, orchestrating inflammatory and immune responses through interactions with various receptors.^[^ ^20^ ^]^ Our investigation revealed that the pyroptotic microenvironment induced the formation of MET. To elucidate the underlying molecular mechanisms governing MET generation within this pyroptotic environment, we exposed macrophages to pyroptotic‐CM and assessed the activation of signaling pathways via western blot analysis. Our findings confirmed that the pyroptotic‐CM elicited MET formation through p38 MAPK/p65 NF‐kB signaling cascades. Furthermore, examination of downstream signaling related to HMGB1 indicated that inhibition of TLR4 using a specific inhibitor prevented activation of the p38 MAPK/p65 NF‐kB pathway and reduced MET generation. This suggests that MET formation within the pyroptotic microenvironment occurs via the HMGB1‐TLR4‐p38 MAPK/p65 NF‐kB pathway.

The maintenance of mucosal homeostasis necessitates the precise regulation of epithelial barrier functionality, which is partially contingent upon the presence of barrier‐forming components within the epithelium and the equilibrium between inflammatory and anti‐inflammatory elements in the mucosa.^[^ ^21^ ^]^ Increasing evidences suggest that damage of the intestinal barrier function significantly contributes to the progression of HAEC.^[^ ^22^ ^]^ The epithelial barrier serves as a dynamic and selective membrane that facilitates nutrient absorption; however, it remains susceptible to various biochemical insults due to its direct exposure to pathogens and food‐borne antigens.^[^ ^23^ ^]^ The formation of METs is regarded as a pro‐inflammatory event. Previous studies have demonstrated the involvement of METs in various pathological conditions, including kidney damage resulting from rhabdomyolysis, hepatic ischemia/reperfusion injury associated with iron overload, and phagocytosis by Candida albicans.^[^ ^24^ ^]^ Nevertheless, the role of METs in regulating intestinal mucosal immunity remains largely unexplored and warrants further investigation. In this study, our findings revealed that exposing CECs to METs resulted in decreased cell viability, increased ROS production and pyroptosis, indicating a potential role for METs in damaging the intestinal epithelial barrier during HAEC.

The primary advantage of our research lies in the initial revelation of the crosstalk between pyroptosis and MET formation in HAEC. METs further amplify inflammation and induce damage to CECs, thereby contributing to the progression of HAEC. However, this study has certain limitations. Although LPS transfection is a classical and widely recognized method for triggering pyroptosis, there exist alternative approaches to elicit this process, each characterized by distinct underlying mechanisms.^[^ ^25^ ^]^ Supplementary investigations are necessary to systematically evaluate whether MET formation is sensitive to the microenvironment released by proptotic macrophages induced via distinct methods and diverse agents with differing concentration gradients and temporal parameters. This would enable a more comprehensive characterization of the underlying mechanisms involved. Furthermore, the molecular mechanisms by which the pyroptotic microenvironment induces MET formation and how METs lead to injury in CECs require further validation in a mouse model of HAEC.

In conclusion, our study suggests a functional model wherein HMGB1 derived from pyroptotic cells activates macrophages and induces MET formation via TLR4‐p38 MAPK/p65 NF‐kB pathway. Furthermore, DAMPs in METs lead to decreased cell viability, increased ROS production, and pyroptosis of CECs. These findings reveal a previously unidentified mechanism of cellular communication among pyroptotic cells, macrophages and CECs in HAEC, potentially providing new avenues for therapeutic intervention.

## 4 Experimental Section

### Tissue Specimens

Human paraffin‐embedded colon sections from HAEC patients and healthy control subjects were obtained from the Department of Clinical Nutrition, Shanghai Children's Medical Center, Shanghai Jiao Tong University School of Medicine. The use of colon tissue samples from patients in this study was approved by the joint committee of ethics of the Shanghai Children's Medical Center (SCMC) affiliated to Shanghai Jiao Tong University School of Medicine (SCMCIRB‐K2012022). The colon tissues of mouse model were from our previous studies.^[^ ^10^ ^]^

### Mice

C57BL/6 mice were from Shanghai SLAC Laboratory Animal Co. Ltd (Shanghai, China). All procedures for mice were performed in accordance with the relevant laws and guidelines of the Institutional Animal Care and Use Committee (IACUC) of Shanghai Jiao Tong University (SCMC‐LAWEC‐2021‐0).

### Cell Culture

The RAW264.7, iBMDM, and CT26 cells used in this study were obtained from Cell Bank, Chinese Academy of Sciences. All cell lines were cultured in medium following the manufacturer's protocol. All cells were confirmed to be mycoplasma‐free using a mycoplasma‐detecting test (YEASEN, Shanghai).

### Generation of BMDMs

The femurs and tibias were harvested from the WT mice, followed by the bone marrow being flushed with prechilled Dulbecco's modified Eagle's medium (DMEM). Briefly, the cell pellets were collected by centrifugation at 4 °C, and the erythrocytes were lysed with RBC lysis buffer (Thermo Fisher Scientific). The resultant cells were then washed two times with phosphate‐buffered saline (PBS) and suspended in the DMEM containing 10% fetal bovine serum (FBS) complemented with 50µg mL^−1^ penicillin/streptomycin and 10 ng mL^−1^ recombinant macrophage‐colony stimulating factor (Sigma‐Aldrich, St. Louis, MO, USA) at a concentration of 1 × 10^6^ cells mL^−1^ and seeded into 6‐cm ultra‐low attachment surface plates (Corning Costar, Corning, NY, USA). The BMDM culture medium was changed on day 3 and day 5. BMDM were entirely differentiated and ready for use at day 7.

### Preparation of Pyroptotic‑Conditioned Medium

Pyroptotic conditions medium (Pyroptotic‐CM) were prepared when macrophages cultured to 90% confluence and incubated through LPS transfection. The procedure was outlined as follows: First, mix 2 µg of LPS with the transfection reagent. Gradually add the LPS‐reagent mixture to 2 ml of cell culture medium containing macrophages, ensuring that the final concentration reaches 1 µg mL^−1^. Following this, incubate the cells for a duration of 18 h to induce pyroptosis. The supernatant was collected and centrifuged at 500 × g for 10 min at 4 °C to remove cell debris for subsequent experiments.

### Treatment of Macrophages

Macrophages were incubated with normal‐CM, pyroptotic‐CM or recombinant HMGB1 protein for 4h. For experiments that used inhibitors, macrophages were pretreated with inhibitors to the addition of pyroptotic‐CM. The inhibitors used in this study included an HMGB1 antagonist (HY‐N0184, MCE, USA), a p38 MAPK pathway inhibitor (HY‐12839, MCE, USA), a p65 NF‐kB pathway inhibitor (HY‐138537, MCE, USA), an ERK pathway inhibitor (HY‐112287, MCE, USA), a JNK inhibitor (HY‐12041, MCE, USA), a TLR2 antagonist (HY‐112146, MCE, USA), a TLR4 antagonist (HY‐11109, MCE, USA), a TLR9 antagonist (HY131952, MCE, USA), and a RAGE antagonist (HY‐P2268).

### Wound Healing Assays

For the wound healing assay, iBMDM cells were digested and seeded in 6‐well plates with medium containing 10% FBS. When cells were ≈90% confluent, a 200 µL sterile pipette tip was used to scratch the cell surface, followed by three washes with PBS. Thereafter, the cells were incubated in medium supplemented with or without cell‐free METs. Images of the scratch were acquired at different time points under a microscope. The cell migration rate was calculated as follows: (width at 0 h‐width at different time points)/width at 0 h.

### METs Detection

To evaluate the amount of METs, the levels of extracellular DNA were explored by fluorometry.^[^ ^26^ ^]^ The macrophages were cultured in 96‐well plates at a concentration of 10^6^ cells mL^−1^. At the indicated time points after treatment, 1 U mL^−1^ micrococcal nuclease (New England Biolabs, Ipswich, MA) was added. Macrophages were incubated at 37 °C for 15 min to allow the extruded DNA to detach from the cell debris. Cells were then centrifuged at 1800 g for 10 min. Cell‐impermeable DNA‐binding dye SYTOX Green (Thermo Fisher Scientific, Waltham, MA) was added to the extracted supernatants and incubated in the dark for 15 min. Extracellular DNA content was represented by the mean fluorescence intensity (MFI) detected with SpectraMax M2 (excitation wavelength 485 nM and emission wavelength 530 nM).

Flow cytometry was also used to analyze the proportion of METs forming cells.^[^ ^27^ ^]^ The macrophages were made to react with a plasma membrane‐impermeable DNA‐binding dye, SYTOX Green (Life Technologies) according to the manufacturer's instruction. After filtering out the debris with a mesh, the percolated cells were analyzed using BD flow cytometer. Because SYTOX Green expresses fluorescence only after binding to DNA, the step to remove unbound dye can be omitted.

Histone‐DNA complexes were recognized as precise indicators of MET formation and can be quantified using ELISA methodology.^[^ ^28^ ^]^ In this experimental procedure, macrophages were subjected to the previously described treatment protocol. To prepare for MET detection, macrophages were treated with 1 U mL^−1^ micrococcal nuclease (New England Biolabs, Ipswich, MA) at 37 °C for a 15‐min incubation period, facilitating the separation of extruded DNA from cellular remnants. The resulting supernatant was then collected and centrifuged at 1800 g for 10 min to remove residual cellular debris. For the ELISA setup, 96‐well immuno‐plates were prepared by coating them with anti‐histone antibodies that adhered overnight at 4 °C in a specialized coating buffer. Following this step, the plates underwent a blocking process using a solution of 3% BSA in PBS. Subsequently, aliquots of the prepared sample solution (100 µL) were added to the plates and incubated overnight at 37 °C. Detection of histone‐DNA complexes was accomplished using peroxidase‐conjugated anti‐DNA POD obtained from the Cell Death Detection ELISA kit (Roche, catalog number: 11544675001). The enzymatic reaction was visualized through the application of TMB substrate; quantitative analysis was performed by measuring optical densities (ODs) at a wavelength of 405 nm while utilizing a reference wavelength of 490 nm using a SUNRISE microplate reader (Tecan).

### Detection of HMGB1 in Supernatant by Enzyme‐Linked Immunosorbent Assay (ELISA)

HMGB1 levels in the culture supernatant were quantified using an ELISA. Following the experimental protocol, macrophages were cultured on glass slides and subjected to the specified treatments. Subsequently, the supernatant was collected for analysis. HMGB1 quantification was performed utilizing a commercial ELISA kit (Shanghai Jianglai Biotechnology, JL13702), strictly adhering to the manufacturer's instructions. Optical density measurements were conducted at a wavelength of 450 nm using a multimode microplate reader (TECAN) for accurate determination of HMGB1 concentrations.

### Western Blot Assay

Western blotting was performed as previously described. Briefly, proteins were extracted from cells using RIPA lysis buffer containing proteinase inhibitors. Equal amounts of protein (20 µg) were separated by 10% or 12% SDS‐PAGE. Following electrophoresis, proteins were transferred to a PVDF membrane, blocked in 5% nonfat milk, and incubated overnight at 4°C with the following primary antibodies: caspase‐1, GSDMD, citH3, IL‐1B, ERK1/2, p‐ERK1/2, p65 NF‐kB, p‐p65 NF‐kB, p38 MAPK, p‐p38 MAPK, JNK, p‐JNK and GAPDH. After washing with TBST three times, the membrane was incubated with HRP‐conjugated goat anti‐rabbit or anti‐mouse secondary antibodies (Bioworld Technology) at RT for 2 h. The protein bands were visualized by enhanced chemiluminescence and analyzed with ImageJ software (National Institutes of Health, Bethesda, MD, USA). GAPDH served as the loading control.

### Immunofluorescence Staining

Macrophages were seeded and incubated on glass sides and treated according to the experimental design. To detect and quantify METs, the samples were incubated overnight at 4 °C with citrullinated histone H3 (citH3; Abcam, ab5103) and CD68(Abmart, MN50019). Cells were then incubated with Alexa Fluor 594‐conjugated goat anti‐mouse (1:300, A11032, UK), Alexa Fluor 488‐conjugated goat anti‐rabbit (1:500, Ab150080, UK) and DAPI secondary antibodies. For immunofluorescence staining in vivo, paraffin sections of colon were deparaffinized, antigen‐repaired using sodium citrate buffer, and antigen‐blocked with immunofluorescence blocking buffer (Cell Signaling Technology, 12411). Next, sections were incubated overnight at 4 °C with the following primary antibodies: CD68 (Abmart, MN50019), citrullinated histone H3 (citH3; Abcam, ab5103), caspase‐1 (Abmart, T510200) and HMGB1(Abcam, AB18256). Tissues were then incubated with Alexa Fluor 594‐conjugated goat anti‐mouse (1:300, A11032, UK), Alexa Fluor 488‐conjugated goat anti‐rabbit (1:500, Ab150080, UK) and DAPI secondary antibodies.

### Histology

Colon tissues were fixed in 4% phosphate‐buffered formaldehyde solution for 24 h and embedded in paraffin. Sections of 4 µm were stained with H&E. Colon inflammation and tissue damage were scored based on the degree of epithelial damage and inflammatory infiltrate in the mucosa, submucosa, and muscularis/serosa.

### Quantitative Real‐Time RT PCR (qRT‐PCR)

Total RNA was extracted from BMDM and CT26 cells using TRIzol reagent (Roche, Switzerland), and 1 µg of RNA was reverse transcribed to cDNA by using reverse transcriptase (YEASEN, China). Real‐time quantitative PCR was performed by using a SYBR Green I real‐time detection kit (Thermo Fisher Scientific, USA) on a Bio‐Rad CFX96 Detection System. Relative mRNA expression was normalized to GAPDH expression. The primers used for amplification of the target genes are listed in Table S1 (Supporting Information).

### Preparation of Cell‐Free METs

Macrophage cells were treated with pyroptotic‐CM for 6 h and the supernatant was collected and centrifuged at 500 × g for 10 min at 4 °C to remove cell debris. Then the supernatant was collected for subsequent experiments.

### Cell Viability

Cell viability was detected by Cell Counting Kit 8 (YEASEN) following the manufacturer's instructions. CT26 cells were plated into 96‐well plates and treated with PBS or METs. Next, cells were treated with CCK‐8 and detected by SYNERGY2 microplate reader (BioTek Instruments) at 450 nm. OD value indicated the cell viability.

### ROS Measurement

After CT26 cells were treated in accordance with the experimental design, ROS production was measured by using DCFDA assay kit in accordance with the manufacturer's instructions. Briefly, ≈2 × 10^6 ^cells were harvested, resuspended in PBS supplemented with 2 µM DCFH‐DA (Beyotime, China) reagents and then incubated at 37 °C for 20 min. Then the cells were subjected to fluorometry and flow cytometry analysis at an excitation wavelength of 488 nm and emission wave length of 525 nm.

### RNA‐seq Analysis

For RNA‐seq, BMDM cells were collected after treatment with HMGB1 for 6 h. Total RNA was extracted using the TRIzol reagent (Invitrogen, CA, USA) according to the manufacturer's protocol. Then the libraries were constructed using VAHTS Universal V6 RNA‐seq Library Prep Kit according to the manufacturer's instructions. The transcriptome sequencing and analysis were conducted by OE Biotech Co., Ltd. (Shanghai, China). The libraries were sequenced on an Illumina Novaseq 6000 platform and 150 bp paired‐end reads were generated. The clean reads were mapped to the reference genome using HISAT2. Differential expression analysis was performed using the DESeq2. Q value < 0.05 and foldchange > 2 or foldchange < 0.5 was set as the threshold for significantly differential expression gene (DEGs). The data of RNA sequencing were available in the SRA Database (https://www.ncbi.nlm.nih.gov/sra/PRJNA1185191).

### Statistical Analysis

The data were analyzed using Prism version 9.0 (GraphPad Software Inc). P values were calculated using a paired or unpaired two‐tailed Student's t test for 2 groups and one‐way ANOVA for multiple groups. Correlation analysis was calculated using the Pearson correlation coefficient. The details of data presentation, sample size (n) for each statistical analysis, and statistical methods were demonstrated in the indicated figure legends. P values < 0.05 were considered to indicate statistical significance.

## Conflict of Interest

The authors declare no conflicts of interests.

## Author Contributions

Z.Z. and L.H. conceived the study. R.Z. and J.L. performed experiments. L.S., L.P., and C.Z. provided critical advice. R.Z. and Z.Z. wrote paper.

## Supporting information



Supporting Information

## Data Availability

The data that support the findings of this study are available from the corresponding author upon reasonable request.
