# Supplementary material for: HMGB1 Derived from the Pyroptotic Microenvironment Promotes Macrophage Extracellular Traps in Hirschsprung‐Associated Enterocolitis
Source: Adv Biol (Weinh). 2025 Jun 4;9(9):e00761. doi: 10.1002/adbi.202400761 (PMC12447116; doi:10.1002/adbi.202400761)
Supplement: Supplementary file 1 — Supporting Information [file ADBI-9-e00761-s001.docx]

**HMGB1 Derived from the Pyroptotic Microenvironment Promotes Macrophage Extracellular Traps in Hirschsprung-Associated Enterocolitis**

*Rui Zhang, Jing Li, Lili Song, Liya Pan, Chengchen Zhang, Zhiyan Zhan^*^ and Li Hong^*^*

**Supplementary Table 1. The sequences of primers for qPCR (mouse)**

| Gene | Sequence |
| --- | --- |
| iNOS | F: 5'-CAGATCGAGCCCTGGAAGAC-3'  R: 5'-CTGGTCCATGCAGACAACCT-3' |
| IL-1B | F: 5'-TGCCACCTTTTGACAGTGATG-3'  R: 5'-AAGGTCCACGGGAAAGACAC-3' |
| TNFa | F: 5'-CATCTTCTCAAAATTCGAGTGACAA-3'  R: 5'-TGGGAGTAGACAAGGTACAACCC-3' |
| CCL2 | F: 5'-TCATGCTTCTGGGCCTGCTGT-3'  R: 5'-CTCATTGGGATCATCTTGCTGGTG-3' |
| CCL5 | F: 5'-CCACTCCCTGCTGCTTTGCCTA-3'  R: 5'-TGGCACACACTTGGCGGTTC-3' |
| Mapkapk2 | F: 5'-TCGACAAGAGAACCCAGCAA-3'  R: 5'-CTCCACCATCGAGACACTCC-3' |
| Met | F: 5'-AGCTGACGGTGTAGCAGAAC-3'  R: 5'-CGTGAAGTTGGGGAGCTGAT-3' |
| Cd40 | F: 5'-TTGTTGACAGCGGTCCATCT-3'  R: 5'-TCTCAAGAGCTGTGCAGTGG-3' |
| Cxcl10 | F: 5'-TGCCGTCATTTTCTGCCTCA-3'  R: 5'-AGGCTCGCAGGGATGATTTC-3' |
| Cxcl2 | F: 5'-AGGGCGGTCAAAAAGTTTGC-3'  R: 5'-CAGGTACGATCCAGGCTTCC-3' |
| Cxcl3 | F: 5'-GAAAGGAGGAAGCCCCTCAC-3'  R: 5'-ACACATCCAGACACCGTTGG-3' |
| Icam1 | F: 5'-TGTCAGCCACCATGCCTTAG-3'  R: 5'-CAGCTTGCACGACCCTTCTA-3' |
